# Prevalence and associated factors of overweight in Chinese adolescents: A cross‐sectional study

**DOI:** 10.1002/hsr2.2237

**Published:** 2024-07-04

**Authors:** Junjie Huang, Vera M. W. Keung, Calvin K. M. Cheung, Amelia S. C. Lo, Sze C. Chan, Yuet Y. Wong, Lancelot W. H. Mui, Albert Lee, Martin C. S. Wong

**Affiliations:** ^1^ Centre for Health Education and Health Promotion, Faculty of Medicine The Chinese University of Hong Kong Hong Kong SAR; ^2^ Jockey Club School of Public Health and Primary Care, Faculty of Medicine The Chinese University of Hong Kong Hong Kong SAR; ^3^ The School of Public Health Peking University Beijing China; ^4^ The School of Public Health The Chinese Academy of Medical Sciences and The Peking Union Medical Colleges Beijing China; ^5^ The School of Public Health Fudan University Shanghai China

**Keywords:** adolescents, obesity, overweight, risk factors, students

## Abstract

**Background and Aim:**

Obesity has been a global public health issue due to the increasing mortality rate and prevalence among children. However, there are scarce studies on obesity prevalence in Hong Kong children. The study aims to identify the risk factors of obesity among primary and secondary school students by assessing the relationship between sociodemographic factors, health‐related behaviors, and social relationships.

**Methods:**

Self‐administrated surveys were collected from 30 primary schools and 25 secondary schools participating in the “Quality Education Fund Thematic Network on Health Schools” project. Descriptive analysis was conducted to examine the proportions of different characteristics and to compare the disparity between primary and secondary school students with obesity.

**Results:**

A total of 4884 responses were collected. A larger proportion of primary school students with obesity were male (adjusted odds ratio [aOR]: 2.55, 95% confidence interval [CI]: 1.77–3.67, *p* < 0.001) and actively gamed (aOR: 1.64, 95% CI: 1.07–2.51, *p* = 0.024). Secondary school students with obesity were male (aOR: 1.61, 95% CI: 1.21–2.13, *p* = 0.001), had poor self‐perceived academic performance (aOR:1.51, 95% CI: 1.10–2.08, *p* = 0.011) and expressed higher life satisfaction (family) (aOR: 1.13, 95% CI: 1.01–1.26, *p* = 0.032). There were negative associations found between obesity and physical activity, high consumption of sugary drinks, chocolate or candies, and insufficient consumption of vegetables.

**Conclusion:**

Male sex, physical inactivity, low self‐perecived academic performance, and poor dietary behaviors were the risk factors for obesity among primary and secondary school students. The findings highlighted the importance of identifying younger individuals who were at risk of becoming clinically obese. Further studies should explore the effectiveness of various interventions through longitudinal study.

## 1 INTRODUCTION

Obesity is a global health issue in the 21st century. There are more than 1.9 billion adults, aged 18 years or above, who are overweighted and over 650 million who are obesed.^1^, ^2^, ^3^ A total of 39 million children below the age of five reported having health issues caused by obesity.^1^ According to the World Health Organization (WHO), obesity has killed more people than malnourishment in countries where the majority of the world's population is living.^1^, ^2^

Growth standards and health assessment have been developed by the Department of Health to detect obesity in the youth population, specifically targeting primary and secondary school students in Hong Kong.^4^, ^5^ Obesity is defined as having a body weight of more than 120% of the median value of weight for height. This is used to assess male students who are between 55 cm and 175 cm tall and female students who are 55 cm and 165 cm tall, with a body mass index (BMI) larger than 25.^4^ Rates of obesity have increased from 16.4% to 21.4% and 12.6% to 18.7% among primary school students and secondary school students respectively from the 1997/1998 school year to the 2010/2011 school year.^5^, ^6^

Compared to children of a healthy weight, overweight children reported more negative experiences and significant practical and psycho‐social problems. For example, poor health status and diseases, low self‐esteem, poor school performance, and a higher risk of being bullied.^6^, ^7^ This could also result in poor health status and reduced employment and marital prospects in adulthood.^8^, ^9^

Promoting well‐balanced diets and regular exercise amongst students, schools, and parents has been shown to help in preventing obesity.^1^, ^2^, ^3^, ^5^, ^6^ Healthy behaviors may include 60 min of moderate to high‐intensity physical activities every day, controlling sugar and fat intake, and sufficient intake of vegetables and fruits.^5^, ^6^ Education about maintaining a healthy weight has been suggested to target families with children who are at risk of obesity as indicated by high BMI values or high birth weight.^6^ It is important to investigate the risk factors leading to obesity among children to provide early prevention.^10^, ^11^, ^12^ Early preventive strategies have been evaluated in a study involving 64 German kindergartens. Positive behavioral changes were observed as vegetable and fruit consumption increased among children.^12^, ^13^

However, studies regarding the prevalence of obesity in children are still limited in Hong Kong.^14^, ^15^ This study aims to identify the risk factors of obesity among primary and secondary school students by assessing the relationship between sociodemographic factors, health‐related behaviors, and their social relationships. This helps identifiying individuals who are at risk of obese at an early stage and develops preventive measures targeting high‐risk adolescents.

## 2 METHODOLOGY

### 2.1 Participant recruitment

This study recruited students from local primary and secondary schools participating in the project “Quality Education Fund Thematic Network on Health Schools.” The project was carried out by the Centre for Health Education and Health Promotion of the Chinese University of Hong Kong (CHEHP). A total of 30 primary schools and 25 secondary schools participated in this project.

### 2.2 Data collection

There were several procedures for survey collection. First, students were informed that participation was voluntary and data collection would be anonymized to protect privacy of the students. Students were assured that their responses would be confidential and their grades would remain unaffected by their participation or responses. Second, parental consent and consent from the school were collected before conducting the survey. Third, training was provided to the interviewers who were responsible for informing students about the purpose and features of the survey. Fourth, the confidentiality of the data collected was emphasized. Class teachers helped to identify students who had parental consent to participate in the study. However, they were not involved in answering student queries and did not have access to the questionnaire responses.

### 2.3 Survey instruments

The survey was validated using pilot tests and approved by expert panels consisting of epidemiologists, healthcare professionals, and physicians. There were 19 questions in the survey, consisting of Traditional Chinese and English version. Sociodemographic information including sex, age, number of household computers, ownership of cars (household), ownership of private bedroom, traveling history in the past 12 months, self‐perceived academic performance, and self‐reported expectations from parents (Questions 1–5) were collected from participants. Health‐related parameters including the presence of mental health outcomes such as psychological distress, depression, and self‐harm, weight and height, weight control, and frequency of physical activity of at least moderate intensity were examined. Only secondary school students reported the quality of their sleep (Questions 6–13). Social relationships were assessed using the number of friends, the experience of being bullied at school and online, and the life satisfaction level of family and friends (Questions 14–17). Lastly, participants were required to report experiences of drinking alcohol and smoking (Questions 18–19). A 5‐point Likert scale has been used in the survey. The detailed questionnaire has been provided in Supporting Information S1: Material 1.

### 2.4 Variables

In this study, obesity was defined according to the assessment guide of childhood growth and nutrition published in 1995 (reference). Students were classified into “overweight,” “normal range,” and “underweight” according to their sex, height, and weight. Overweight refers to those whose weight exceeds 120% of the median of the weight and height status; normal range refers to those who weigh between 80% and 120% of the median, and underweight refers to those whose weight is below 80% of the median.^16^ If the height of boys or girls exceeds 175 cm or 165 cm respectively, BMI cutoff points for the Asian population were used for classification. For example, the range of 18.5–22.9 kg/m^2^ would be classified as normal weight.^17^

The Family Affluence Scale (FAS) was used to measure socioeconomic status. Psychological distress of mental health outcomes was measured by using the Kessler Psychological Distress Scale (K6) with a cutoff score of 13.^18^, ^19^ Depression was defined as a prolonged feeling of despair whereas having any intention, plans, attempts or experience in injuring themselves or committing suicide was counted as self‐harm. The Pittsburgh Sleeping Quality Index (PSQI) was used to evaluate sleep quality. Detailed coding of the variables has been listed in Supporting Information S1: Material 2.

### 2.5 Statistical analysis

IBM Statistical Package for Social Sciences (SPSS) software version 26.0 was used to conduct statistical analyses. The descriptive analysis examined the characteristics of the study participants including the disparity between the primary and secondary school students. The difference in respondents' characteristics was presented in proportions with the outcome variables. Separate binary regression models were designed to examine the association between the risk factors and outcome variables (overweight). Also, multiple logistic regression models were used to examine the explanatory association between factors and the outcome variables after adjustment. All *p*‐values less than 0.05 were considered statistically significant.

## 3 RESULTS

### 3.1 Respondents' characteristics

A total of 4884 responses were collected with 2234 (45.7%) reponses from primary school students and 2650 (54.3%) responses from secondary school students. The overall prevalence of obesity was 17.3% and 16.8% for primary and secondary school students respectively. The sex proportions of the two groups were largely balanced, with 49.5% (*n* = 1310) primary school cohort and 51.6% (*n* = 1146) of the secondary school cohort being male. Significant variations in socioeconomic status, self‐perceived academic performance, and parental expectation were observed across the two groups. Students in the primary school cohort reporting better socioeconomic status (medium or high: 81.6%, vs. 71.3%, *p* < 0.001), better self‐perceived academic performance (average to good: 80.3%, vs. 62.7%, *p* < 0.001) and higher expectation from parents (high: 57.4%, vs. 43.2%, *p* < 0.001) than the secondary school cohort. Secondary school students had a higher prevalence of physical inactivity than primary school studnets (28.1% vs. 11.3%, *p* < 0.001) They also spent more time on gaming, more than 2 h during weekdays (47.8% vs. 18.8%, *p* < 0.001) and social media (40.0% vs. 6.7%, *p* < 0.001) than primary school students. Regarding mental health conditions, although the proportions of students experiencing depression and self‐harm tendencies were comparable among the two groups, students in secondary school had a significantly higher prevalence of psychological distress (18.6% vs. 10.0%, *p* < 0.001). In terms of social relationships, secondary school students were more vulnerable to bullying online (16.9% vs. 13.4%, *p* < 0.001), whilst primary school students were significantly more likely to have experienced being bullied at school (53.6% vs. 33.9%, *p* < 0.001). As far as dietary habit is concerned, secondary school students were significantly more likely to consume more desserts, ice cream, cakes, or tarts (13.9% [consumed at least four times a week], vs. 11.3% in primary school, *p* = 0.005), chocolate or candies (22.4% vs. 17.6%, *p* < 0.001), soft drinks (19.0% vs. 9.2%, *p* < 0.001), carton‐packed juice, lemon tea, or other sugary drinks (37.5% vs. 20.6%, *p* < 0.001), and processed or preserved meat (19.7% vs. 14.3%, *p* < 0.001). There are significantly moresecondary school students skipped breakfast at least once a week (54.2% vs. 17.4%, *p* < 0.001), and did not have at least one serving of vegetables (63.7% vs. 56.5%, *p* < 0.001) or fruits (54.1% vs. 29.9%, *p* < 0.001). Detailed characteristics of the respondents of the two groups can be found in Table 1.

**Table 1:** Participant characteristics.

	Primary school	Secondary school	*p*‐Value
Sex			
Male	1146 (51.6%)	1310 (49.5%)	0.163
Female	1077 (48.4%)	1334 (50.5%)	
Socioeconomic status			
Low	397 (18.4%)	751 (28.8%)	<0.001*
Medium	1130 (52.5%)	1359 (52.1%)	
High	627 (29.1%)	500 (19.2%)	
Academic performance			
Poor	431 (19.7%)	987 (37.4%)	<0.001*
Average	925 (42.3%)	868 (32.9%)	
Good	831 (38.0%)	787 (29.8%)	
Expectation from parents			
Low	91 (4.2%)	140 (5.3%)	<0.001*
Average	839 (38.5%)	1360 (51.5%)	
High	1252 (57.4%)	1139 (43.2%)	
Sleeping quality			
Good quality		1302 (55.1%)	N/A
Poor quality		1059 (44.9%)	
Moderate physical activity (number of days)			
Not at all	244 (11.3%)	739 (28.1%)	<0.001*
1–4 Days	1251 (57.8%)	1541 (58.6%)	
5–7 Days	668 (30.9%)	350 (13.3%)	
More than 2 h of … during weekdays			
Gaming			
No	1795 (81.2%)	1371 (52.2%)	<0.001*
Yes	415 (18.8%)	1256 (47.8%)	
Social media			
No	2048 (93.3%)	1577 (60.0%)	<0.001*
Yes	146 (6.7%)	1052 (40.0%)	
Depression			
No	1451 (70.6%)	1828 (70.0%)	0.658
Yes	604 (29.4%)	783 (30.0%)	
Self‐harm			
No	1747 (81.4%)	2094 (80.3%)	0.348
Yes	400 (18.6%)	514 (19.7%)	
Psychological distress			
No	1939 (90.0%)	2135 (81.4%)	<0.001*
Yes	215 (10.0%)	489 (18.6%)	
Experience of being bullied			
No	1019 (46.4%)	1734 (66.1%)	<0.001*
Yes	1176 (53.6%)	890 (33.9%)	
Experience of being cyberbullied			
No	1877 (86.6%)	2182 (83.1%)	<0.001*
Yes	291 (13.4%)	445 (16.9%)	
Unhealthy food consumption			
Crisps and other snacks			
No	1936 (87.2%)	2348 (88.9%)	0.074
Yes	284 (12.8%)	294 (11.1%)	
Chocolate or candies			
No	1825 (82.4%)	2048 (77.6%)	<0.001*
Yes	389 (17.6%)	591 (22.4%)	
Desserts, ice‐cream, cake, or tart			
No	1964 (88.7%)	2267 (86.1%)	0.005*
Yes	249 (11.3%)	367 (13.9%)	
Soft drinks			
No	1999 (90.8%)	2136 (81.0%)	<0.001*
Yes	203 (9.2%)	500 (19.0%)	
Carton‐packed juice, lemon tea, or other sugary drinks			
No	1749 (79.4%)	1644 (62.5%)	<0.001*
Yes	453 (20.6%)	985 (37.5%)	
Fried food			
No	1988 (90.1%)	2334 (88.5%)	0.086
Yes	219 (9.9%)	302 (11.5%)	
Processed or preserved meat			
No	1888 (85.7%)	2117 (80.3%)	<0.001*
Yes	316 (14.3%)	520 (19.7%)	
Regular breakfast			
Yes	1722 (82.6%)	1149 (45.8%)	<0.001*
No	363 (17.4%)	1362 (54.2%)	
At least one serving of			
Vegetables			
Yes	805 (43.5%)	833 (36.3%)	<0.001*
No	1047 (56.5%)	1462 (63.7%)	
Fruits			
Yes	1358 (70.1%)	1102 (45.9%)	<0.001*
No	579 (29.9%)	1297 (54.1%)	

### 3.2 Prevalence of overweight among patients with various characteristics

Overall, male primary school students had a significantly higher prevalence of being overweight (22.0% vs. 12.4%, *p* < 0.001). Furthermore, a higher prevalence of overweight was found among primary school students who had poor self‐perceived academic performance (24.6% vs. 14.2%–16.3% [good to average], *p* < 0.001), spent more than 2 h on gaming (24.6% vs. 15.7%, *p* < 0.001) and social media (23.9% vs. 16.9%, *p* = 0.038) during weekdays, had self‐harm tendencies (21.1% vs. 16.1%, *p* = 0.021), had experiences of being bullied at school (20.2% vs. 14.3%, *p* < 0.001) or online (21.5% vs. 16.5%, *p* = 0.044), and did not have regular breakfast (24.6% vs. 15.1%, *p* < 0.001). Additionally, students who consumed more carton‐packed juice, lemon tea, or other sugary drinks (22.4% vs. 16.2%, *p* = 0.003), fried food (25.0% vs. 16.6%, *p* = 0.003), and processed or preserved meat (24.7% vs. 16.2%, *p* < 0.001) were more likely to be overweight.

Among secondary school students, a higher prevalence of obesity was associated with the male sex (19.7% vs. 14.0%, *p* < 0.001), and poor self‐perceived academic performance (20.0% vs. 14.8%–14.9%, *p* = 0.003). On the contrary, students who consumed more crisps and other snacks (12.6% vs. 17.4%, *p* = 0.042), chocolate or candies (11.5% vs. 18.4%, *p* < 0.001), desserts, ice‐cream, cakes or tarts (12.2% vs. 17.5%, *p* = 0.012), sugary drinks (14.6% vs. 18.2%, *p* = 0.018) were significantly less likely to be overweight. The full list of characteristics by outcome can be found in Table 2.

**Table 2:** Prevalence of overweight among students with various characteristics.

	Primary school		Secondary school	
	*n* (Prevalence %)	*p*‐Value	*n* (Prevalence %)	*p*‐Value
Total	363 (17.3%)		162 (16.8%)	
Sex				
Female	127 (12.4%)	<0.001*	183 (14.0%)	<0.001*
Male	236 (22.0%)		247 (19.7%)	
Socioeconomic status				
Low	70 (18.9%)	0.265	141 (19.2%)	0.106
Medium	193 (18.3%)		202 (15.5%)	
High	92 (15.4%)		82 (16.8%)	
Academic performance				
Good	112 (14.2%)	<0.001*	113 (14.8%)	0.003*
Average	142 (16.3%)		125 (14.9%)	
Poor	97 (24.6%)		190 (20.0%)	
Expectation from parents				
High	206 (17.4%)	0.325	182 (16.5%)	0.645
Average	127 (16.3%)		219 (16.6%)	
Low	19 (22.6%)		26 (19.7%)	
Sleeping quality				
Good quality			214 (16.9%)	0.542
Poor quality			163 (16.0%)	
Moderate physical activity (number of days)				
Not at all	48 (21.5%)	0.157	134 (18.6%)	0.294
1–4 days	197 (16.7%)		242 (16.3%)	
5–7 days	101 (16.1%)		52 (15.4%)	
More than 2 h of … during weekdays				
Gaming				
No	268 (15.7%)	<0.001*	209 (15.7%)	0.133
Yes	93 (24.6%)		216 (17.9%)	
Social media				
No	325 (16.9%)	0.038*	249 (16.4%)	0.576
Yes	32 (23.9%)		176 (17.2%)	
Depression				
No	231 (16.8%)	0.445	309 (17.4%)	0.236
Yes	103 (18.3%)		116 (15.5%)	
Self‐harm				
No	265 (16.1%)	0.021*	346 (17.1%)	0.636
Yes	79 (21.1%)		81 (16.2%)	
Psychological distress				
No	309 (17.0%)	0.225	335 (16.3%)	0.177
Yes	41 (20.4%)		90 (18.8%)	
Experience of being bullied				
No	139 (14.3%)	<0.001*	269 (16.0%)	0.171
Yes	220 (20.2%)		155 (18.1%)	
Experience of being cyber bullied				
No	291 (16.5%)	0.044*	350 (16.6%)	0.498
Yes	59 (21.5%)		78 (18.0%)	
Unhealthy food consumption				
Crisps and other snacks				
No	312 (17.1%)	0.288	394 (17.4%)	0.042*
Yes	51 (19.8%)		36 (12.6%)	
Chocolate or candies				
No	297 (17.3%)	0.809	363 (18.4%)	<0.001*
Yes	65 (17.9%)		66 (11.5%)	
Desserts, ice‐cream, cake or tart				
No	319 (17.2%)	0.346	385 (17.5%)	0.012*
Yes	44 (19.7%)		43 (12.2%)	
Soft drinks				
No	318 (16.9%)	0.052	352 (17.0%)	0.434
Yes	42 (22.6%)		75 (15.6%)	
Carton‐packed juice, lemon tea, or other sugary drinks				
No	268 (16.2%)	0.003*	289 (18.2%)	0.018*
Yes	92 (22.4%)		139 (14.6%)	
Fried food				
No	310 (16.6%)	0.003*	374 (16.5%)	0.276
Yes	51 (25.0%)		55 (19.1%)	
Processed or preserved meat				
No	289 (16.2%)	<0.001*	339 (16.5%)	0.363
Yes	71 (24.7%)		91 (18.2%)	
Regular breakfast				
Yes	246 (15.1%)	<0.001*	187 (16.7%)	0.992
No	82 (24.6%)		218 (16.7%)	
At least one serving of				
Vegetables				
Yes	114 (15.1%)	0.116	161 (20.0%)	<0.001*
No	178 (18.0%)		198 (14.0%)	
Fruits				
Yes	220 (17.1%)	0.672	178 (16.6%)	0.947
No	87 (16.3%)		207 (16.5%)	

### 3.3 Multivariate logistic regression

Among primary school students, the male sex was associated with an increased risk of being overweight (adjusted odds ratio [aOR]: 2.55, 95% confidence interval [CI]: 1.77–3.67, *p* < 0.001). Significant associations were also found between being overweight and more than 2 h of gaming during weekdays (aOR: 1.64, 95% CI: 1.07–2.51, *p* = 0.024), and not having breakfast on a daily basis (aOR: 2.00, 95% CI: 1.32–3.03, *p* = 0.001).

As far as the secondary school group is concerned, a higher risk of being overweight was associated with the male sex (aOR: 1.61, 95% CI: 1.21–2.13, *p* = 0.001), poor self‐perceived academic performance (aOR:1.51, 95% CI: 1.10–2.08, *p* = 0.011), higher life satisfaction (family) (aOR: 1.13, 95% CI: 1.01–1.26, *p* = 0.032), and higher consumption of processed or preserved meat (aOR: 1.49, 95% CI: 1.06–2.11, *p* = 0.023). On the other hand, physical activity of moderate intensity was associated with a lower risk of being overweight (1–4 days: aOR: 0.74, 95% CI: 0.55–0.99, *p* = 0.043; 5–7 days: aOR: 0.52, 95% CI: 0.33–0.83, *p* = 0.006, compared with no physical activity at all). It was found that higher consumption of chocolate or candies (aOR: 0.60, 95% CI: 0.41–0.88, *p* = 0.008) and sugary drinks (aOR: 0.58, 95% CI: 0.42–0.79, *p* = 0.001), and insufficient consumption of vegetables (aOR: 0.60, 95% CI: 0.45–0.79, *p* < 0.001) were negatively associated with overweight risk. Full results (aORs and their 95% CIs) of the regressions are included in Table 3.

**Table 3:** Factors associated with overweight based on multivariable logistic regression.

	Primary school			Secondary school		
	aOR	95% CI	*p*‐Value	aOR	95% CI	*p*‐Value
Sex						
Female	1 (ref)			1(ref)		
Male	**2.546**	**1.767–3.669**	<0.001*	**1.605**	**1.208–2.134**	0.001*
FAS score						
Low	1 (ref)			1 (ref)		
Average	0.805	0.522–1.242	0.327	**0.739**	**0.549–0.995**	0.047*
High	0.672	0.409–1.105	0.117	0.831	0.571–1.210	0.334
Academic performance						
Excellent or good	0.801	0.549–1.168	0.249	1.003	0.717–1.403	0.986
Average	1 (ref)			1 (ref)		
Bad or Poor	1.264	0.793–2.015	0.324	**1.512**	**1.098–2.083**	0.011*
Expectation from parents						
Very high or high	1.073	0.746–1.542	0.704	0.927	0.708–1.214	0.581
Average	1 (ref)			1 (ref)		
Low or very low	1.541	0.651–3.646	0.326	0.553	0.278–1.102	0.092
Sleeping quality						
Good quality				1 (ref)		
Poor quality				1.079	0.666–1.750	0.757
Moderate Physical activity (Number of days)						
Not at all	1 (ref)			1 (ref)		
1–4 day(s)	0.912	0.534–1.558	0.736	**0.738**	**0.550–0.990**	0.043*
5‐–7 days	0.760	0.416–1.388	0.372	**0.523**	**0.329–0.834**	0.006*
More than 2 h of … during weekdays (ref: no)						
Gaming	**1.637**	**1.066–2.514**	0.024*	1.037	0.794–1.356	0.788
Social media	1.059	0.529–2.118	0.872	0.951	0.720–1.256	0.723
Mental health conditions (ref: no)						
Psychological distress	0.840	0.461–1.531	0.570	1.284	0.873–1.889	0.203
Depression	0.984	0.650–1.490	0.941	0.730	0.515–1.034	0.077
Self‐harm	1.115	0.704–1.766	0.643	1.093	0.764–1.565	0.626
Experience of being bullied (ref: no)						
At school	1.125	0.778–1.627	0.531	1.070	0.804–1.424	0.641
Online	1.177	0.725–1.911	0.509	1.256	0.890–1.771	0.195
Life satisfaction						
Family	1.007	0.890–1.140	0.911	**1.127**	**1.010‐1.257**	0.032*
Friends	0.929	0.821–1.053	0.249	0.926	0.826‐1.039	0.191
Unhealthy food consumption						
Crisps or other snacks	0.999	0.575–1.735	0.997	1.076	0.678–1.709	0.755
Chocolate or candies	0.917	0.567–1.483	0.724	**0.598**	**0.408–0.876**	0.008*
Desserts, ice‐cream…	0.914	0.492–1.700	0.777	0.872	0.557–1.366	0.551
Soft drinks	0.657	0.343–1.260	0.206	1.098	0.754–1.600	0.626
Sugary drinks	1.021	0.660–1.579	0.925	**0.576**	**0.419–0.792**	0.001*
Fried food	1.317	0.718–2.415	0.374	1.488	0.967–2.288	0.071
Processed or preserved meat	1.231	0.752–2.016	0.409	**1.492**	**1.057–2.108**	0.023*
At least one serving of… (ref: yes)						
Vegetables	1.181	0.834–1.673	0.348	**0.597**	**0.454–0.785**	<0.001*
Fruits	0.700	0.473–1.036	0.074	1.161	0.886–1.520	0.278
Regular breakfast (ref: yes)						
No	**1.997**	**1.317–3.028**	0.001*	1.105	0.842–1.450	0.472

Abbreviations: aOR, adjusted odds ratio; CI, confidence interval.

## 4 DISCUSSION

### 4.1 Summary of major findings

The results of this study found that male sex, poor self‐perceived academic performance, and higher sugar and fat intake were the main characteristics of both primary and secondary students who had a higher prevalence of obesity. Compared with primary school students, secondary school students were more active in gaming and social media and inactive in physical activity. They were more likely to have an unhealthy diet, such as having more sugar and fat intake and fewer vegetables or fruits, and to experience psychological distress. It has been found that primary school students who were active in gaming and had a behavior of skipping breakfast were more likely to be overweight. Among secondary school students, physical inactivity, satisfaction with family, and consumption of processed or preserved meat were the causes of obesity, while those who had an unhealthy diet of high‐consuming sugar drinks, chocolate, or candies and insufficient consumption of vegetables were less likely to be overweight.

### 4.2 Risk factors associated with overweight or obesity among students

The sex difference between overweight and obesity among students has been proven. Studies have found that the increase in obesity rates in boys was greater than that in girls, by 151% and 81%, respectively.^10^, ^20^ Across 188 countries, there were 65% of the countries reported that a higher prevalence of obesity has been found in boys aged 5–9 years than those in girls, and 60% of countries reported a similar trend among those aged 10–19 years.^21^ Similar results have been found in different studies and countries including Canada, China, and Poland.^21^, ^22^, ^23^, ^24^, ^25^ The differences might be due to biological differences in body composition between sexes and sociocultural influences. For example, leptin level which helped suppress appetite and promote energy utilization was higher among females than males.^21^, ^26^ Besides, gender‐based stereotypes also required females to eat less, and be healthier and thin. Therefore, girls would have more weight‐related concerns than boys, especially in high‐income countries.^21^, ^27^, ^28^ It was common that male students were more likely to have obesity issues.

The poor academic result was also a key characteristic of secondary school students who are overweight. A study based on a national youth risk behavior survey has found that youth aged 14–17 years and classified as overweight were less likely to report higher grades (OR 0.79, 95% CI: 0.68–0.91, *p* = 0.002). The result has shown that being overweight was significantly related to academic performance.^28^ Another study also found a significant association between overweight status and poorer school performance. Children who were teased about their weight barely had strong school performance.^29^ A study that examined the relationship between nutritional status and academic performance of primary school students aged 9–10 years old in Malaysia has found that academic performance was correlated with BMI, income and educational level of their parents, and whether they have taken breakfast.^30^ However, another study in Kuwait did not find any association between obesity and academic performance in the classroom setting among 1066 male students.^31^ The relationship between overweight and obesity and academic performance was still critical.

The current study suggested that primary school students, in general, had better socioeconomic status, self‐perceived academic performance, and higher self‐reported expectations from parents which are similar to a previous study where the prevalence of overweight and obesity was examined in 6729 children and significant differences were reported between the highest and the lowest SES group.^32^ Children who were from the lowest SES group which was measured by the index of community socio‐educational advantage (ICSEA) based on parents' occupation and education, school geographical location and the proportion of indigenous students had a lower level of educational advantage, and had a higher prevalence of overweight and obesity among schools. It has been suggested that children from lower SES backgrounds had lower levels of physical activity, higher consumption of fast food and sugar‐sweetened soft drink, and were more difficult to receive and actively respond to health promotion messages. The results of these studies implied that healthy lifestyle promotion should consider the complex interaction among individual, family, school as well as community factors reflected by socioeconomic status.^32^, ^33^, ^34^

Certain dietary patterns are positively associated with overweight and obesity among children.^35^ WHO has reported the increased consumption of certain foods such as refined carbohydrates, sugar‐rich food, bakery products, and food of animal origin. The main reasons for increasing obesity in many countries were due to the dependence on sweets and soft drinks and the reduced intake of vegetables and fruits.^35^, ^36^ Although studies have found that consumption of non‐obesogenic foods such as vegetables, fruits, whole grains, and nuts that were classified as healthy, less sugary, and fatty food was less risky to have overweight or obese, they also mentioned dietary patterns could be varied according to the cultural and economic context, and other factors such as lifestyle and level of physical activity.^35^, ^36^, ^37^ Diet should be viewed within a complex context with lifestyle and health, especially when living standards were rising in many countries.^38^ Our studies have found a different result that higher consumption of sweets and sugary drinks was negatively associated with the risk of being overweight. There were also other studies reflecting that children who had an obesogenic diet did not necessarily present the risk of obesity.^35^, ^39^, ^40^ Similar to the above studies, some studies suggested that unhealthy food consumption should be examined overall with health‐related behaviors among children, for example, the behavior of consuming takeaway, being allowed to consume snacks anytime, and receiving sweet rewards.^41^, ^42^

Findings regarding dietary patterns and unhealthy food consumption were varied according to the ways of study even though it was common to find that a higher intake of fat, sugar, and carbohydrate tended to have a higher risk of being overweight due to higher caloric intake.^41^ However, the findings regarding physical inactivity as a critical factor of overweight and obesity were undoubted. In many studies, a positive association has been found between physical inactivity and being overweight, for example, the odds ratio was 3.36 (95% CI 1.68–6.72) or 1.6 (OR 1.6, 95% CI 1.1–2.4) respectively in two studies.^43^, ^44^ Insufficient physical activity may include watching videos and television and doing homework for more than 2 h, which consequently increases the risk of obesity. This study has identified the difference in physical activity levels between primary school and secondary school students. Secondary school students spent more time on gaming and social media than primary school students. This was similar to the findings of other studies. When children get older, they tend to spend more time playing video games, sitting in front of the computer, or watching television and become less active. A high decline in physical activity may also take place during adolescence at the age of 15–18.^45^, ^46^, ^47^

Although psychological issues did not find significantly related to being overweight in the present study, it had appeared to be an important factor since there were almost 1 billion children, between the ages of 2–17 years old, who had experienced physical, emotional, or sexual violence globally.^48^, ^49^ This study shown that the experiences of being bullied among primary and secondary school students such as in school and online were associated with a higher prevalence of obesity. Regardless the type of violence, students who had these experiences tended to report psychological distress and long‐term physical or mental health issues. Additionally, these students were also more vulnerable to developing reactive attachment disorder, report low levels of physical inactivity and be at risk of obesity.^49^

### 4.3 Limitations

There are a few limitations of the current study that should be noted. First, there was a potential clustering effect as the survey was done school‐based that students within the same age group and the same school may share similar characteristics. Hence, the result may be overrepresented or underrepresented within a cluster. Second, there had been a slight disparity in the geographical distribution of the participants between our study and Hong Kong population (Supporting Information S1: Table 1). The prevalence of overweight among secondary school students might have been overestimated due to the lower proportion of participants from Hong Kong Island. This was because significant association was found between overweight and socioeconomic status, yet, residents in Hong Kong Island had a better socioeconomic status. Furthermore, generalizing the findings of this study to other populations should be done meticulously due to the nonrandom sampling adopted to collect the data and the uneven responses of each school. Additionally, due to the cross‐sectional design of the study, the causal relationship was not evaluated as the time series of the occurrence of the two events cannot be identified. Nevertheless, the association identified in this study provided the foundation for future longitudinal studies to explore the risk factors as causal factors.

## 5 CONCLUSION

The current study identified individuals with a higher risk of being overweight by multivariable logistic regression. Factors associated with being overweight included the male sex, physical inactivity, poor self‐perceived academic performance, and unhealthy dietary habits. Hence, multifactorial intervention such as immediate and intensive lifestyle modification is crucial. Future studies should explore the effectiveness of various interventions after examining the associations with a longitudinal study.

## AUTHOR CONTRIBUTIONS

**Junjie Huang**: Conceptualization; writing—original draft. **Vera M. W. Keung**: Data curation; formal analysis. **Calvin K. M. Cheung**: Data curation; formal analysis. **Amelia S. C. Lo**: Data curation; formal analysis. **Sze Chai Chan**: Data curation; formal analysis; writing—original draft. **Yuet Yan Wong**: Writing—review & editing. **Lancelot W. H. Mui**: Writing—review & editing. **Albert Lee**: Writing—review & editing. **Martin C. S. Wong**: Writing—review & editing; conceptualization.

## CONFLICT OF INTEREST STATEMENT

The authors declare no conflict of interest.

## ETHICS STATEMENT

Survey and Behavioral Research Ethics (no. SBRE(R)−22‐008), The Chinese University of Hong Kong, Hong Kong SAR.

## TRANSPARENCY STATEMENT

The lead author Martin C. S. Wong affirms that this manuscript is an honest, accurate, and transparent account of the study being reported; that no important aspects of the study have been omitted; and that any discrepancies from the study as planned (and, if relevant, registered) have been explained.

## Supporting information

Supporting information.

## Data Availability

The data that support the findings of this study are available from the corresponding author upon reasonable request. The data sets used and/or analyzed during the current study are available from the corresponding author on reasonable request.
