# Supplementary material for: Prevalence and associated factors of overweight in Chinese adolescents: A cross‐sectional study
Source: Health Sci Rep. 2024 Jul 4;7(7):e2237. doi: 10.1002/hsr2.2237 (PMC11224025; doi:10.1002/hsr2.2237)
Supplement: Supplementary file 1 — Supporting information. [file HSR2-7-e2237-s001.pdf]

Supplementary legends

**Supplementary Table 1**	Geographical distribution of participants
**Supplementary material 1**	Questionnaire
**Supplementary material 2**	Coding of variables

Supplementary Table 1 Geographical distribution of participants

	Hong Kong Population^	Primary school	Secondary school
New Territories	3984077 (53.7%)	1115 (49.9%)	1527 (57.6%)
Kowloon	2232339 (30.1%)	968 (43.3%)	848 (32.0%)
Hong Kong Island	1195529 (16.1%)	151 (6.8%)	275 (10.4%)

^According to 2021 Hong Kong Population Concensus

Supplementary material 1 Questionnaire

1. What is your gender?

2. What is your age?

3a. Does your family own any cars or other vehicles?

No

Yes

Yes, two or above

3b. Do you have your own bedroom? (not including shared room with family members)

Yes

No

3c. Have you travelled outside Hong Kong with your family over the past 12 months? (does not include visiting relatives in your hometown or paying tribute to ancestors)

No

Once

Twice

Three times or more

3d. How many computers do you have at home? (desktop computers, laptops and tablets)

Zero

One

Two

Three or more

4. How would you describe your academic performance in the past 12 months? (compared with other students at your grade)

Excellent

Good

Average

Bad

Poor

5. What level of expectation do you think your parents have on your academic performance?

Very high

High

Average

Low

Very low

The following questions are about your emotional status and experiences of self-harm. Your response will never be accessed by anyone other than the researchers.

6. ***In the past 30 days****, how often did you feel:*

6a. nervous?

None of the time

A little of the time

Some of the time

Most of the time

All of the time

6b. hopeless?

None of the time

A little of the time

Some of the time

Most of the time

All of the time

6c. restless or fidgety?

None of the time

A little of the time

Some of the time

Most of the time

All of the time

6d. so depressed that nothing could cheer you up?

None of the time

A little of the time

Some of the time

Most of the time

All of the time

6e. that everything was a burden?

None of the time

A little of the time

Some of the time

Most of the time

All of the time

6f. worthless?

None of the time

A little of the time

Some of the time

Most of the time

All of the time

*7. **In the past 12 months**, did you ever feel so sad or hopeless almost every day for two weeks or more/*

Never

Yes

*8a. **In the past 12 months**, did you ever intentionally injure yourself? (e.g. bruising yourself or skin-cutting)*

Yes

No

*8b. **In the past 12 months**, did you ever seriously consider attempting suicide?*

Yes

No

*8c. **In the past 12 months**, did you actually attempt suicide?*

Yes

No

9. What is your weight?

10. What is your height?

*11. **In the past 7 days**, on how many days did you have physical activity of moderate or vigorous intensity for a total of at least 60 minutes per day?*

Not at all

1-4 day(s)

5-7 days

12a. In general, how many hours, in total, do you spend on playing videos games or computer games?

On normal school days:

12b. In general, how many hours, in total, do you spend on using social networking Apps or social websites? For example, Facebook, Instagram, WhatsApp, WeChat, or Blogs.

On normal school days:

The following questions relate to your usual sleep habits during the past month only. Your answer should indicate the most accurate reply for the majority of days and nights in the past month.

13a. During the past month, what time have you usually gone to bed at night?

9:00 pm

9:30 pm

10:00 pm

10:30 pm

11:00 pm

11:30 pm

12:00 am

12:30 am

1:00 am

1:30 am

Other specific time:

*13b. **During the past month**, how long (in minutes) has it usually taken you to fall asleep each night?*

0 to 15 minutes

16 to 30 minutes

31 to 60 minutes

Over 60 minutes

*13c. **During the past month**, what time have you usually gotten up in the morning?*

5:00 am

5:30 am

6:00 am

6:30 am

7:00 am

7:30 am

8:00 am

8:30 am

Other specific time:

13d. ***During the past month****, how many hours of actual sleep did you get at night? This may be different than the number of hours you spent in bed.*

About 5 hours

About 5.5 hours

About 6 hours

About 6.5 hours

About 7 hours

About 7.5 hours

About 8 hours

About 8.5 hours

About 9 hours

Other specific time:

13e. ***During the past month****, how often have you had trouble sleeping because you… (not during the past month, less than once a week, once or twice a week, three or more times a week)*

Cannot get to sleep with 30 minutes

Wake up in the middle of the night or early morning

Have to get up to use the bathroom

Cannot breathe comfortably

Cough or snore loudly

Feel too cold

Feel too hot

Had bed dreams

Have pain

13f. Besides the reasons aforementioned, are there any other reasons that cause you having trouble sleeping? If yes, please specific the reason: _____________________

How often during the past month have you had trouble sleeping because of this?

Not during the past month

Less than once a week

Once or twice a week

Three or more times a week

*13g. **During the past month**, how would you rate your sleep quality overall?*

Very good

Fairly good

Fairly bad

Very bad

*13h. **During the past month**, how often have you taken medicine to help you sleep? (prescribed or "over the counter")?*

Not during the past month

Less than once a week

Once or twice a week

Three or more times a week

The following questions are about being bullied. Your response will never be accessed by anyone other than the researchers.

*On school property: **In the past 30 days**, did/were you (Yes/ No)*

14a. being beaten by schoolmates?

14b. being socially isolated, teased or gossip by schoolmates (e.g. name-calling)?

14c. Frightened by words or actions?

14d. Found your personal belongings being damaged?

14e. Sexually harassed (Sex-related languages, moves, bodily contacts, acts and written or electronic messages that made you feel insulted, disgusted, frightened, uncomfortable or threatened)?

*On the Internet: **In the past 30 days**, did/were you (Yes/ No)*

15a. find your personal information being disclosed without your permission?

15b. receive offensive or insulting comments?

15c. find someone spreading gossips about you?

15d. find someone using your e-mail or social networking accounts to play practical joke?

15e. receive threatening or frightening messages?

The following questions are about your view of life. Please respond according to your true feelings instead of according to expectations from others or yourself.

Please choose the most appropriate description for your satisfaction with the following aspects:

16a. Family life

Terrible

Unhappy

Mostly dissatisfied

Mixed (Equally satisfied & dissatisfied)

Mostly satisfied

Pleased

Delighted

16b. Friendship

Terrible

Unhappy

Mostly dissatisfied

Mixed (Equally satisfied & dissatisfied)

Mostly satisfied

Pleased

Delighted

The following questions are about your dietary habits. Please respond according to your true feelings instead of according to expectations from others or yourself.

***During the past 7 days**, how often did you eat:*

17a. Crisps or other snacks (e.g. potato chips, prawn crackers) (~35 grams per packet)

No

1-3 times in 7 days

4-6 times in 7 days

Once or more a day

17b. Chocolate or candies (3 to 5 pieces)

No

1-3 times in 7 days

4-6 times in 7 days

Once or more a day

17c. Desserts, ice-cream, cake or tart (~150 grams per piece or cup)

No

1-3 times in 7 days

4-6 times in 7 days

Once or more a day

17d. Soft drinks (~330 mL per can)

No

1-3 times in 7 days

4-6 times in 7 days

Once or more a day

17e. Carton-packed juice, lemon tea or other sugary drinks (~250mL per carton)

No

1-3 times in 7 days

4-6 times in 7 days

Once or more a day

17f. Fried food (e.g. French fries, fried chicken, etc.)

No

1-3 times in 7 days

4-6 times in 7 days

Once or more a day

17g. Processes or preserved meat (e.g. sausage, ham, BBQ pork, Chinese sausage, etc.)

No

1-3 times in 7 days

4-6 times in 7 days

Once or more a day

18a. ***In the past 7 days****, how much vegetables did you eat every day?*

Less than one serving

At least one serving

18b. ***In the past 7 days****, how much fruit did you eat every day? One serving of fruit approximately equals to the portion of 1 medium-sized fruit (e.g. kiwifruit), half large-sized fruit (e.g. dragon fruit) or half bowl of fruit (e.g. grapes).*

Less than one serving

At least one serving

*19****. In the past 7 days****, on how many days did you eat breakfast?*

Every day

5 to 6 days

3 to 4 days

1 to 2 days

0 days

Not sure

Supplementary material 2 Coding of variables

Only variables undergone transformation are shown.

VAR3a. Does your family own any cars or other vehicles?

No (0)

Yes (1)

VAR3b. Do you have your own bedroom? (not including shared room with family members)

No (0)

Yes (1)

VAR3c. Have you travelled outside Hong Kong with your family over the past 12 months? (does not include *visiting relatives in your hometown or paying tribute to ancestors)*

No (0)

Once (1)

Twice (1)

Three times or more (1)

VAR3d. How many computers do you have at home? (desktop computers, laptops and tablets)

Zero (0)

One (1)

Two (1)

Three or more (1)

Description: VAR3a-3d has been transformed into a binary categorical data to indicate the possession of any computer at home.

VAR4. How would you describe your academic performance in the past 12 months? (compared with other students at your grade)

Excellent (0)

Good (0)

Bad (1)

Poor (1)

Description: VAR4 has been transformed into a binary categorical data to indicate the possession of any computer at home.

VAR5. What level of expectation do you think your parents have on your academic performance?

Very high (0)

High (0)

Average (1)

Low (2)

Very low (2)

Description: VAR5 has been transformed into continuous data to indicate the possession of any computer at home.

VAR6. In the past 30 days, how often did you feel: (None of the time (0), a little of the time (1), some of the time (2), most of the time (3), all of the time (4))

6a. nervous?

6b. hopeless?

6c. restless or fidgety?

6d. so depressed that nothing could cheer you up?

6e. that everything was a burden?

6f. worthless?

VAR6: Psychological distress: Presence (13-24), Absence (0-12)

Description: VAR6 based on the K6 scale,[1] the total score is the summation of the scores of the 6 items. The total score is 24, and the cut-off is 13.[2]

8a. In the past 12 months, did you ever intentionally injure yourself? (e.g. bruising yourself or skin-cutting)

Yes (1)

No (0)

8b. In the past 12 months, did you ever seriously consider attempting suicide?

Yes (1)

No (0)

8c. In the past 12 months, did you actually attempt suicide?

Yes (1)

No (0)

VAR8: Self-harm: Yes (1), No (0)

Description: VAR8a-8c have been combined, any (1) of the 4 questions of 8a-8c leads to an overall (1) in VAR8.

VAR12a. In general, how many hours, in total, do you spend on playing videos games or computer games?

Less than 2 hours (0)

2 hours or more (1)

VAR12b. In general, how many hours, in total, do you spend on using social networking Apps or social websites? For example, Facebook, Instagram, WhatsApp, WeChat, or Blogs.

Less than 2 hours (0)

2 hours or more (1)

Description: VAR12a and 12b have been transformed into a binary categorical data to indicate the possession of any computer at home.

The following questions relate to your usual sleep habits during the past month only. Your answer should indicate the most accurate reply for the majority of days and nights in the past month.

13a. During the past month, what time have you usually gone to bed at night?

13b. During the past month, how long (in minutes) has it usually taken you to fall asleep each night?

< 15 minutes (0)

16-30 minutes (1)

31-60 minutes (2)

> 60 minutes (3)

13c. During the past month, what time have you usually gotten up in the morning?

13d. During the past month, how many hours of actual sleep did you get at night? This may be different than the number of hours you spent in bed.

> 7 hours (0)

6-7 hours (1)

5-6 hours (2)

< 5 hours (3)

13e. During the past month, how often have you had trouble sleeping because you… (not during the past month (0), less than once a week (1), once or twice a week (2), three or more times a week (3))

i. Cannot get to sleep with 30 minutes

ii. Wake up in the middle of the night or early morning

iii. Have to get up to use the bathroom

iv. Cannot breathe comfortably

v. Cough or snore loudly

vi. Feel too cold

vii. Feel too hot

viii. Had bed dreams

ix. Have pain

13f. Besides the reasons aforementioned, are there any other reasons that cause you having trouble sleeping? If yes, please specific the reason.

How often during the past month have you had trouble sleeping because of this?

Not during the past month (0)

Less than once a week (1)

Once or twice a week (2)

Three or more times a week (3)

13g. during the past month, how would you rate your sleep quality overall?

Very good (0)

Fairly good (1)

Fairly bad (2)

Very bad (3)

13h. During the past month, how often have you taken medicine to help you sleep (prescribed or "over the counter")?

Not during the past month (0)

Less than once a week (1)

Once or twice a week (2)

Three or more times a week (3)

13i. During the past month, how often have you had trouble staying awake while travelling, eating meals, or engaging in social activity/

Not during the past month (0)

Less than once a week (1)

Once or twice a week (2)

Three or more times a week (3)

13j. During the past month, how much of a problem has it been for you to keep up enough enthusiasm to get things done?

Not during the past month (0)

Less than once a week (1)

Once or twice a week (2)

Three or more times a week (3)

VAR13a – Component 1: Subjective sleep quality (Q13g)

VAR13b – Component 2: Sleep latency (sum of Q13b and Q13e(i), sum: 0 = component 2: 0; sum: 1-2, component 2: 1; sum: 3-4, component 2: 2; sum: 5-6, component 2: 3).

VAR13c – Component 3: Sleep duration (Q13d)

VAR13d – Component 4: Sleep efficiency (Q13d / (sum of Q13a + Q13c + Q13d)) * 100%)

> 85% (0); 75-84% (1); 65-74% (2); < 65% (3)

VAR13e – Component 5: Sleep disturbance (sum of Q13e(ii~ix) and Q13f)

Sum: 0 (0); 1-9 (1); 10-18 (2); 19-27 (3)

VAR13f – Component 6: Use of sleep medication (Q13h)

VAR13g – Component 7: Daytime dysfunction (sum of Q13i and Q13j, sum: 0 = component 7: 0; sum: 1-2, component 7: 1; sum: 3-4, component 7: 2; sum: 5-6, component 7: 3).

VAR13h: Sum of (VAR13a to VAR13g), 5 or above: poor sleep quality; less than 5: good sleep quality

Description: The question 13 was based on the Pittsburgh Sleep Quality Index (PSQI).[3] VAR13c has been used as a continuous variable for sleep duration. VAR12h has been used as a binary categorical variable for sleep quality with a cut-off at 5.[4]

16. Please choose the most appropriate description for your satisfaction with the following aspects:

(Terrible (1), Unhappy (2), Mostly dissatisfied (3), Mixed (Equally satisfied & dissatisfied) (4), Mostly satisfied (5), Pleased (6), Delighted (7))

16a. Family life

16b. Friendship

Description: VAR16a and 16b have been used as a continuous variable.

17. The following questions are about your dietary habits. Please respond according to your true feelings instead of according to expectations from others or yourself.

During the past 7 days, how often did you eat: (No [No/ 1-3 times in 7days] (0), Yes [4-6 times in 7 days/ once or more a day] (1))

17a. Crisps or other snacks (e.g. potato chips, prawn crackers) (~35 grams per packet)

No (0)

1-3 times in 7 days (0)

4-6 times in 7 days (1)

Once or more a day (1)

17b. Chocolate or candies (3 to 5 pieces)

No (0)

1-3 times in 7 days (0)

4-6 times in 7 days (1)

Once or more a day (1)

17c. Desserts, ice-cream, cake or tart (~150 grams per piece or cup)

No (0)

1-3 times in 7 days (0)

4-6 times in 7 days (1)

Once or more a day (1)

17d. Soft drinks (~330 mL per can)

No (0)

1-3 times in 7 days (0)

4-6 times in 7 days (1)

Once or more a day (1)

17e. Carton-packed juice, lemon tea or other sugary drinks (~250mL per carton)

No (0)

1-3 times in 7 days (0)

4-6 times in 7 days (1)

Once or more a day (1)

17f. Fried food (e.g. French fries, fried chicken, etc.)

No (0)

1-3 times in 7 days (0)

4-6 times in 7 days (1)

Once or more a day (1)

17g. Processes or preserved meat (e.g. sausage, ham, BBQ pork, Chinese sausage, etc.)

No (0)

1-3 times in 7 days (0)

4-6 times in 7 days (1)

Once or more a day (1)

Description: VAR17a-17g has been transformed into a binary categorical data to indicate the possession of any computer at home.

19. In the past 7 days, on how many days did you eat breakfast?

Every day (0)

5 to 6 days (1)

3 to 4 days (1)

1 to 2 days (1)

0 days (1)

Not sure (1)

VAR19: Every day = Yes (0), Others = no (1)

Description: VAR19 has been transformed into a binary categorical data to indicate having any experience of travelling.

1. Kessler RC, Andrews G, Colpe LJ, et al. Short screening scales to monitor population prevalences and trends in non-specific psychological distress. *Psychol Med* 2002; **32**(6): 959-76.
2. Kim G, DeCoster J, Bryant AN, Ford KL. Measurement Equivalence of the K6 Scale: The Effects of Race/Ethnicity and Language. *Assessment* 2016; **23**(6): 758-68.
3. Buysse DJ, Reynolds CF, 3rd, Monk TH, Berman SR, Kupfer DJ. The Pittsburgh Sleep Quality Index: a new instrument for psychiatric practice and research. *Psychiatry Res* 1989; **28**(2): 193-213.
4. Buysse DJ, Hall ML, Strollo PJ, et al. Relationships between the Pittsburgh Sleep Quality Index (PSQI), Epworth Sleepiness Scale (ESS), and clinical/polysomnographic measures in a community sample. *J Clin Sleep Med* 2008; **4**(6): 563-71.
